# The adaptive value of phenotypic plasticity in two ecotypes of a marine gastropod

**DOI:** 10.1186/1471-2148-10-333

**Published:** 2010-10-28

**Authors:** Johan Hollander, Roger K Butlin

**Affiliations:** 1 Department of Animal and Plant Sciences, University of Sheffield, Sheffield, N10 2TN, UK

## Abstract

**Background:**

Few surveys have concentrated on studying the adaptive value of phenotypic plasticity within genetically-distinct conspecific ecotypes. Here, we conduct a test to assess the adaptive value that partial phenotypic plasticity may have for survival in the marine gastropod *Littorina saxatilis*. This species has evolved canalized ecotypes but, nevertheless, the ecotypes show some phenotypic plasticity for the traits under divergent selection between wave-exposed and high-predation habitats.

**Results:**

We exposed juveniles of each ecotype to several environmental treatments under laboratory conditions in order to produce shape variation associated with plasticity. The two ecotypes from different treatments were then transplanted to the wave-exposed habitat and the survival rate was monitored. Ecotype explained the largest distinction in survival rate while treatment caused variation in survival rate within the ecotype released into its parental habitat which was correlated with plastic changes in shell shape. Snails that had experienced a treatment mimicking the environment of the transplantation location survived with the highest rate, while individuals from the contrary experimental treatment had lower survivorship.

**Conclusions:**

We conclude that the partial plastic response shown in *Littorina saxatilis *has a significant impact on fitness, although this remains small compared to the overall adaptive difference between ecotypes.

## Background

Phenotypic plasticity is the ability of a single genotype to produce different phenotypes in distinct environments [1-4]. The resulting phenotypic flexibility may increase an organism's fitness in a heterogeneous environment and phenotypic plasticity is a common feature in nature across many taxa [5-9]. However, an organism that encounters an environment that fluctuates over time and/or space may also respond by genetically-based local adaptation, resulting in canalized phenotypes suited to different parts of its range [10].

Phenotypic plasticity can operate jointly with ecotype formation and may further increase fitness. Moderate levels of phenotypic plasticity may facilitate a population's expansion into novel environments as the trait may place the population on the slope of an adaptive peak from which natural selection can advance [11]. Habitats may also change slightly over generations and partial phenotypic plasticity may fine-tune the phenotype around a mean appropriate to the focal habitat. We use the term 'partial plasticity' to refer to cases where the plastic response can explain only a limited proportion of the phenotypic difference between individuals occupying different environments, i.e. cases where genetically distinct ecotypes exist but their phenotypes may be adjusted by plastic responses.

Whereas sensitivity to environmental variation is often an advantage, its adaptive value cannot automatically be assumed [12]. Several cases of adaptive plasticity have been proposed [6,13-15], while there are other examples demonstrating maladaptive plasticity [16]. Some theories posit that a phenotypically plastic response may be able to produce a less extreme phenotype, compared to a canalized phenotype adapted to the same environment, due to the costs of possessing a plastic response or constraints on the ability of plastic development to achieve the target, optimal phenotype (the extreme-phenotype hypothesis) [17,18]. Accordingly, a key step in the study of phenotypic plasticity is to test the putative adaptive value that plasticity may have, preferably by implementing reciprocal transplant experiments in natural conditions [3,19]. This has rarely been attempted for cases of partial plasticity (but see: [20-22]).

*Littorina saxatilis *is a marine gastropod which has gained attention in the study of ecological speciation [23-25]. The species includes two adjacent ecotypes living under different ecological conditions, with one ecotype experiencing predation from the green crab, *Carcinus maenas*, while the other habitat lacks the green crab but is exposed to strong hydrodynamic forces. In Sweden, the conditions with crab predation have selected the S-ecotype (sheltered) to evolve a relatively large and thick shell with a pronounced apex and with a small aperture to prevent winkling [26]. In the absence of crabs but with exposure to wave action a small E-ecotype (exposed) has evolved with a squat morphology and a thin shell, and with a large aperture to increase the foot area and so improve adhesion [27]. Previous studies have utilized transplant experiments on this species and demonstrated that the ecotypes are highly adapted to their native environments as each ecotype showed high mortality in the contrasting habitat [28]. However, despite the genetically-based local adaptation of the ecotypes [29,30], the species shows some plasticity. This is ecotype and habitat specific, with the greatest plasticity in the resident habitat [31]. Ecotype formation is a response to well-understood divergent selection pressures from the crabs and the wave environment. Thus, *Littorina saxatilis *is an excellent model for the study of partial plasticity, especially for a test of the adaptive value of the plastic response within ecotypes, compared to the fitness effects of the genetically-based differences between ecotypes.

Hollander and colleagues [31] demonstrated that the ecotypes of *Littorina saxatilis *are genetically distinct since the plasticity shown in their experiment varied around a mean for each ecotype, but the ecotypes remained well separated despite the variation in environment between treatment groups. Here, we report an experiment where *L. saxatilis *individuals of both ecotypes were exposed to several ecological treatments in a laboratory environment, to initiate plastic development in different ontogenetic directions, and were then transplanted into natural conditions in the wave exposed habitat, to test the adaptive value of any plastic responses that may have influenced phenotypic variation within and between ecotypes. We predicted that the S-ecotype in general would experience high mortality due to fixed maladapted characters, and that the crab-induced form of each ecotype would suffer greater mortality than the wave-exposed form.

## Results

**Figure 1:**
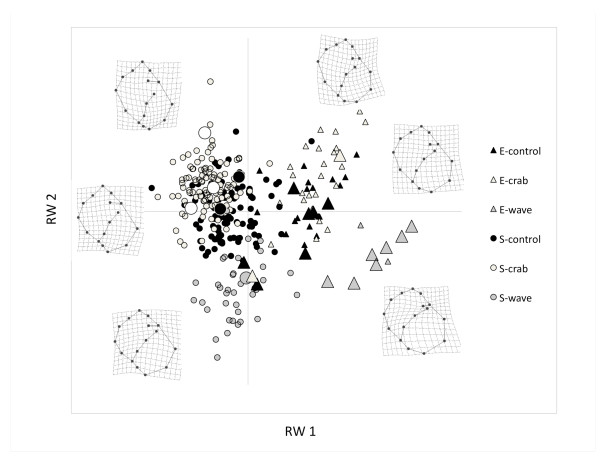
**Shape variation among ecotypes and treatments and its correlation to survival rate**. A relative warp analysis describing the first two axes; RW1 and RW2. Circles illustrate individuals of the S-ecotype while triangles illustrate E-ecotype snails. Shading indicates treatment group. Larger symbols represent individuals that survived the natural transplant experiment. Deformation grids for each ecotype are ordered from the top for "Crab", "Control" and "Wave" treatments in each ecotype.

Following laboratory treatment, snails of the S ecotype were larger than those of the E ecotype (ANOVA: F_1, 293 _= 187.83; P < 0.001) but there was no effect of treatment on size (F_4,293 _= 1.55; P < 0.19). The first two axes of the relative warp analysis described 56.29% of the total variation (RW1 explained 39.81% and RW2 16.49%). The E and S ecotypes were significantly differentiated along the first axis (F_1, 297 _= 823.35; P < 0.001) and treatment groups were discriminated primarily on the second axis (F_2, 297 _= 94.55; P < 0.001) (Figure 1 and Additional file 1). The effect of rearing jar was excluded during model simplification and therefore the F-ratio was tested over the residual variance. *Post hoc *tests found significant differences between all groups (P < 0.001) except for the comparison E-wave vs. E-control. Visualization of deformation grids revealed large variation in relative size around the aperture both between ecotypes and among treatments. The thin-plate spline also demonstrated a deformation zone around the apex with the S-ecotype being more pointed, in general, compared to the E-ecotype.

The numbers of snails that survived from the experimental treatments and were available for transplant to the shore were: 98 S-control, 108 S-crab, 39 S-wave, 25 E-control, 26 E-crab and 9 E-wave (after removing three individuals from each jar for another study, numbers of snails per patch: E-control 5, 5, 6, 6, 3, E-crab: 6, 4, 6, 5, 5, E-wave: 4, 1, 4, S-control 18, 26, 18, 17, 19, S-crab 25, 19, 23, 21, 20 and S-wave 5, 6, 11, 11, 6). Survival rate on the shore differed between ecotypes and treatment groups. Only six snails altogether survived (and were recaptured) among the 245 S-ecotype individuals released. Two individuals survived from the control treatment, 3 from the crab treatment and 1 snail from the wave treatment. The GLM test did not find survival differences among the treatments as expected given such high mortality. Survival of E-ecotype individuals was much higher than S-ecotype individuals (deviance change = 32.58, df = 1, P < 0.001), but varied among treatments: 7 of 9 snails from the wave-simulated treatment survived, 7 of 25 from the control treatment and 2 of 26 from the crab treatment. A GLM testing for an effect of treatment on survival rate among the E-ecotype snails confirmed significant variation among groups (deviance change = 16.35, df = 2, P < 0.001) with snails from the wave-exposed treatment showing significantly higher survival than the other two groups (wave vs. control: z = 2.33, P = 0.020, crab vs. control: z = 1.85, P = 0.064).

The minimum adequate model from the analysis including shape variables eliminated effects of RW1, Patch and Treatment, and retained only a significant effect of the second shape axis, RW2 (deviance change = 18.86, df = 1, P < 0.001, b = -44.8 ± 13.1). Survival increased in the E-ecotype with decreasing RW2 scores, i.e. with more rounded aperture and less pointed spire.

To estimated the strength of selection we included both RW1 and RW2 in the linear regression model and obtained partial regression coefficients (selection gradients *sensu *[32]) of: RW1, 0.184; se 0.052; P < 0.001 and RW2, -0.127; se 0.052; P < 0.023 with 57 degrees of freedom.

## Discussion

We tested the plastic response of the two ecotypes of *Littorina saxatilis *to experimentally altered environments and the consequences of this response for survival in one of the two contrasting natural habitats. The experimental treatments mimicked the major selection pressures experienced by the snails in each habitat, crab predation and hydrodynamic forces due to wave action. We showed that the plastic response over the experimental period is smaller than the difference between ecotypes and in a different direction in shape space. A greater plastic response might have been expected had the snails spent longer in the experimental conditions, and possibly if their mothers had also experienced those conditions. Previous studies have always shown a strong inherited component to the ecotype difference [31], but maternal effects have not been examined. Despite the limited plasticity, the response influenced survival in the expected direction: wave exposed snails of the E-ecotype survived better and crab exposed snails worse, in the wave exposed habitat. The estimated selection gradients were steep, but not atypical for morphological traits [33], both on the axis separating ecotypes (RW1) and on the axis showing the greater plastic response (RW2).

Earlier studies on Swedish *Littorina saxatilis *applying reciprocal transplant experiments have demonstrated a considerable mortality rate in the contrasting habitat [28]. We confirm this result: the mortality rate was very high for all individuals of the S-ecotype, even after experiencing simulated wave exposure, mimicking the environment they were later transplanted into. It is noticeable that the S-ecotype snails remained distant in the morphological shape space from the E-ecotype: the plastic response could not compensate in shell shape sufficiently to increase survival (Figure 1). Our sample size for the E-ecotype that was transplanted to the field was small. Nevertheless, we found significant differences between the groups, both between the ecotypes and also among the experimental groups within the E-ecotype, where shape variation was a strong predictor of survival differences.

Both genetic and plastic variation are pervasive in contributing to species' abilities to evolve local adaptation. Organisms may combine phenotypic plasticity with genetically based fixed characters to increase their average fitness [34,35]. Providing the background environment is stable over generations, the overall phenotype is expected to become more canalized. However, with patches of micro-habitats generating temporal and/or spatial variation, organisms experience some environmental disparity across generations and partial phenotypic plasticity may evolve in concert with the canalized trait to fine-tune the phenotype towards adaptation. A similar study to ours was conducted by Keeley et al. [34] where they examined phenotypic plasticity among ecotype populations of the rainbow trout (*Oncorhynchus mykiss*). The authors discovered that morphological variation among the fish is explained mainly by fixed genetic characters (average 52.7%) while the plasticity response governed only 7.3% or variation. However, for a number of morphological traits, especially among the fins, the balance of control was more in favour of phenotypic plasticity. The authors suggested that this was probably a developmental strategy associated with varying conditions of water flow. They did not test for increased fitness as a result of the plastic responses.

Relyea [36] studied tadpoles of *Rana sylvatica *to test the extreme-phenotype hypothesis and found no relationship between increased plasticity and less extreme phenotypes. This hypothesis is an example of the view that genetically divergent ecotypes and variable phenotypes due to plasticity are opposing strategies to evolve local adaptation. This false dichotomy has hindered the study of combined strategies for adaptive traits (as in the *Littorina *case; canalization of ecotypes and partial phenotypic plasticity), and their effectiveness compared with purely canalized or entirely plastic development. There are, however, several insightful studies in plants that consider phenotypic plasticity among ecotypes [e.g. 37, 38], although few examine explicitly the adaptive value of plasticity within ecotypes (but see: [20-22]).

## Conclusions

Pigliucci [3] stated, *"While ecotypes can indeed be plastic, such plasticity is likely to be either incidental or the residual of previous history"*. Hollander et al. [31] showed that the plastic responses of the E and the S ecotype of *Littorina saxatilis *develop in the same direction, but specific for their parental habitats since the magnitude of the plasticity was greatest in the resident habitat. Our current study clearly demonstrates that these plastic responses are adaptive, at least in one ecotype, likely to be maintained by selection and likely to play a significant role in allowing survival of the snails in contrasting and variable habitats. They are neither incidental nor residual. Studies in plants evaluating the magnitude of plasticity in different ecotypes, the fitness effects that result and the specificity of the phenotypic plasticity [20,21,39,40] similarly show that the observed plasticity is not a relic but an adaptive trait.

In the future, we need to concentrate on the genetic control of developmental strategies to understand, for example, how gene expression varies among ecotypes to produce canalized phenotypes yet simultaneously can be modified in response to environmental cues to influence the same character, albeit in a somewhat different direction. The plasticity observed in this study is most likely not a historical artefact, on the contrary the partial plastic response observed within the E-ecotype of *L. saxatilis *is adaptive and assists the snails to approach a phenotypic optimum.

## Methods

### Collection and laboratory experiments

Snails of the wave-exposed (E-ecotype) and the crab-exposed ecotype (S-ecotype) were sampled during spring 2009 in the archipelago outside Göteborg, Sweden (on the island of Öckerö N 57° 42'49.61 E 11° 37'53.36), at numerous sites and pooled to minimize confounding effects of close relatives. The sampled snails had a size less than 2 mm which means that all snails were recently born, that is, they were of similar age and had been exposed to local environmental effects only for a short time (a few weeks at most).

The two contrasting environments occupied by *Littorina saxatilis *on Swedish rocky coasts have been well-characterised. In the wave-exposed habitat, water motion imposes high levels of hydrodynamic stress [41]. Water motion is mainly wind driven and depends on fetch length, that is the length of water over which a given wind has blown. The water motion causes a hydrodynamic force on the snail proportional to the square of the water velocity and to the area of the object in the direction of the flow, determined by shell size and shape [42,43]. The lift force is opposed by attachment (for gastropods this is most often connected to the foot, and so aperture area). Consequently, a small and a squat shell shape is favoured and is critical for a snail's survival chances in a wave-swept environment [44-46]. The selection regime for *Littorina saxatilis *(the E-ecotype) and other gastropods living in such environments has been extensively studied [44,47-49].

The selection regime for the S-ecotype is different and in many phenotypic traits opposite to the regime for organisms living in strong water motion. Wave action is strongly attenuated on boulder shores. Selection is largely imposed by the green crab (*Carcinus meanas*) which has two main foraging tactics to enter the soft part of the snails, via crushing or pealing the shells, or by "winkling", extracting the soft parts through the shell opening [26,50]. These foraging tactics of the green crab have selected the S-ecotype to evolve protection by means of a thick shell, a narrow aperture and an elongated shell to withdraw the soft parts [49,51-53].

In the laboratory, an experiment with three treatments was conducted, to simulate wave action and the presence of crabs together with a neutral control treatment [31]. The small snails collected from the shore were housed in jars (1 litre), each supplied with sea water at a rate of 1 litre per hour. The treatments are here entitled "Crab", "Wave" and "Control" and each treatment was replicated across five jars for each ecotype, each of which initially contained 30 snails. Jars for the Wave treatment were placed on a rocking table to simulate wave action, with a continuous cycle of period 2hrs generating water motion. These jars, together with the Control treatment jars, were supplied with clean sea water. Jars for the Crab treatment received water containing 'crab effluent', supplied from a separate aquarium that housed several green crabs (a 45 litre aquarium including 10 *Carcinus maenas*). All treatments were kept with a constant water temperature of 8 (±1) degrees Celsius and with a 12 hours light cycle. The experiment was conducted over 90 days, and all snails were then photographed for a landmark-based geometric morphometric analysis [54-56].

### Landmark-based geometric morphometrics

Four fixed anatomical landmarks and twelve sliding-landmarks, describing the curvature on the shell, were applied to capture variation in shell shape. Landmarks was applied on digital images of the shells to describe the overall shell variation and were designed to include the aperture area and the apex area since these are key traits previously shown to be under selection in the contrasting environments. Landmarks and analysis methods are described in full in Hollander et al. [31]. We used the TPS software package http://life.bio.sunysb.edu/morph, tpsDig2 [57] for data acquisition and tpsRelew [58] to perform a generalized Procrustes analysis [59]. The generalized Procrustes analysis excluded the location of the object, rotation and the size of the object, since these produce variation not associated with shape. We obtained 28 shape variables as partial warp scores from the aligned landmark configuration produced by the generalized Procrustes analysis. These shape variables are suitable to employ in traditional multivariate statistical analyses of variation in shape [56,60] and were used here to ordinate the data in a principal component analysis (PCA) as well as in subsequent significance testing.

### Transplant experiment

After photographing, each snail was marked individually with a bee tag glued on the shell and then released in randomly chosen sites on the wave-exposed shore at the island of Öckerö N 57° 42'49.61 E 11° 37'53.36, Göteborg archipelago. This location was selected owing to its exposed position, it is facing westward with a long fetch length into the open sea of Skagerrak. Previous experience of glued bee tags has shown that they reliably stay on the shell for much longer than the short duration of this transplant experiment. All snails were marked in the same way, to ensure equal treatment across groups, and each bee tag had a unique number for individual identification. Each transplant location was denoted as a "Patch" and patch was included in the statistical analysis to control for variation among locations. In each of five patches (only three for E-wave, see Results), snails of one ecotype-treatment combination were placed exposed on the rock without any enclosure within an area of 2m × 2m. Individuals were never placed more than two metres above the sea surface (note that the coast of Sweden lacks tidal oscillation in sea level). The order of patches along the shore was randomized among all treatment groups and the distance between adjacent patches was always larger than six metres, because the cruising range snails is 1-4 metres per 3 months [28]. On the day of releasing the snails, there was no wind or waves, and we made sure that all snails had attached to the substratum as the experiment commenced. We tested survival only in the wave-exposed habitat because previous experience showed that recapture rates are very low in the boulder-fields where crab predation occurs. After 18 days, the snails were counted on the shore to examine the survival rate. During the 18 days of exposure, the snails experienced strong westerly wind speeds up to 20 m/s with a mean of 9.2 m/s (data from SMHI, the Swedish weather agency). Such conditions have the power to produce a water speed in a breaking wave to 5-10 m/s, and in extreme conditions up to 20 m/s, and so to produce strong selection among motile animals in the littoral zone [42,43]. The survival rate measured includes components of both survival and recapture. Because the dispersal rate is low (1-4 m in 3 months; [28]), most snails that were not observed at the end of the experiment are likely to have been dislodged from the rocks in the exposed zone and fallen into deeper water. This almost certainly results in predation by crabs, other invertebrates or fish. Some snails may have been present but not observed but this is likely to be rare because the animals are easily observed on the smooth rocks or in the crevices where they aggregate and there is no reason to expect recapture rate of survivors to vary across treatments in the experiment.

### The statistical analysis

The morphometric analysis produced 28 relative warp components describing the total shell-shape variation of the snails. We included the first two relative warps in a generalized linear model (GLM) in order to examine the association between shell shape and survival. Two categorical variables, Treatment and Patch (nested within Treatment), were also included in the model. Survival was the dependent variable with a binomial state of 0 or 1; the full model with binomial residuals was fitted and simplified by eliminating unnecessary parameters to find the minimum adequate model [61]. We also conducted a GLM analysis excluding the relative warp terms in order to examine the explanatory effect of Treatment regardless of shape. An estimate of the strength of selection (over the duration of the experiment) can be calculated from the relationship between trait values and individual survival [32]. We calculated the selection gradient as the linear regression of fitness (1 for those that survived, 0 for those that did not) on the relative warp scores (RW1 and RW2) obtained from the geometric morphometric analysis, transformed to standard scale. All analysis was conducted using the statistical package R [62].

## Authors' contributions

JH and RKB conceived this study together and the project advanced through close collaboration and discussions.

## Supplementary Material

Additional file 1 **Shape variables**. Shape variables and categorical response variables assessed to describe variation in shell shape among surviving and non-surviving individuals from the two ecotypes and three different laboratory environmental treatments. Click here for file
